# 

**Published:** 1904-09

**Authors:** 


### BOOKS RECEIVED.

The Principles and Practice of Gynecology. For Students and Practitioners. By E. C. Dudley, A. M., M. D., President of the American Gynecological Society; Professor of Gynecology, Northwestern University Medical School, Chicago. Octavo, pp. 771. With 419 illustrations in colors and monochrome, of which 18 are full-page plates. Philadelphia and New York: Lea Brothers & Co. 1904. (Price, cloth, $5.00; leather, $6.00; half morocco, $6.50, net.)

The Theory and Practice of Infant Feeding. With Notes on Development. By Henry Dwight Chapin, A. M., M. D., Professor of Diseases of Children at the New York Post-Graduate Medical School; attending physician to the Post-Graduate, Willard Parker and Riverside Hospitals. Second edition, revised. Octavo, pp. 353. Illustrated. New York: William Wood & Co. 1904.

Radiotherapy and Phototherapy, including Radium and High Frequency Currents. Their Medical and Surgical Applications in Diagnosis and Treatment. By Charles Warrenne Allen, M. D., Professor of Dermatology in the New York Post-Graduate Medical School. Octavo, 618 pages, 131 engravings and 27 plates. Philadelphia and New York: Lea Brothers & Co. 1904. (Cloth, $4.50, net.)

Normal Histology. By Edward K. Dunham, Ph. B., M. D., Professor of General Pathology, Bacteriology and Hygiene in the University and Bellevue Hospital Medical College, New York. New (3d) edition, revised and enlarged. Octavo, 334 pages, with 260 illustrations. Philadelphia and New York: Lea Brothers & Co. 1904. (Cloth, $2.75, net.)

A Textbook of Pathology. By Joseph McFarland, M. D., Professor of Pathology and Bacteriology in the Medico-Chirurgical College of Philadelphia; Pathologist to the Medico-Chirurgical Hospital. Handsome 8vo volume of 818 pages, with 350 illustrations, a number in colors. Philadelphia, New York, London: W. B. Saunders & Co. 1904. (Cloth, $5.00 net; sheep or half morocco, $6.00 net.)

The Optical Dictionary. Edited by Charles Hyatt-Woolf, F. R. P. S., Editor of "The Optician and Photographic Trades Review." Philadelphia: P. Blakiston's Son & Co. 1904. (Price, $1.00.)

The Practical Application of the Röntgen Rays in Therapeutics and Diagnosis. By William Allen Pusey, A. M., M. D., Professor of Dermatology in the University of Illinois; and Eugene W. Caldwell, B. S., Director of the Edward N. Gibbs Memorial *x*-ray Laboratory of the University and Bellevue Hospital Medical College, New York. Second edition, thoroughly revised and enlarged. Handsome 8vo volume of 690 pages, with 195 illustrations, including four colored plates. Philadelphia, New York, London: W. B. Saunders & Co. 1904. (Cloth, $5.00 net; sheep or half morocco, $6.00 net.)

